# Supplementary figures and images for: In vivo multiscale analyses of spring viremia of carp virus (SVCV) infection: From model organism to target species
Source: PLoS Pathog. 2024 Aug 5;20(8):e1012328. doi: 10.1371/journal.ppat.1012328 (PMC11326706; doi:10.1371/journal.ppat.1012328)

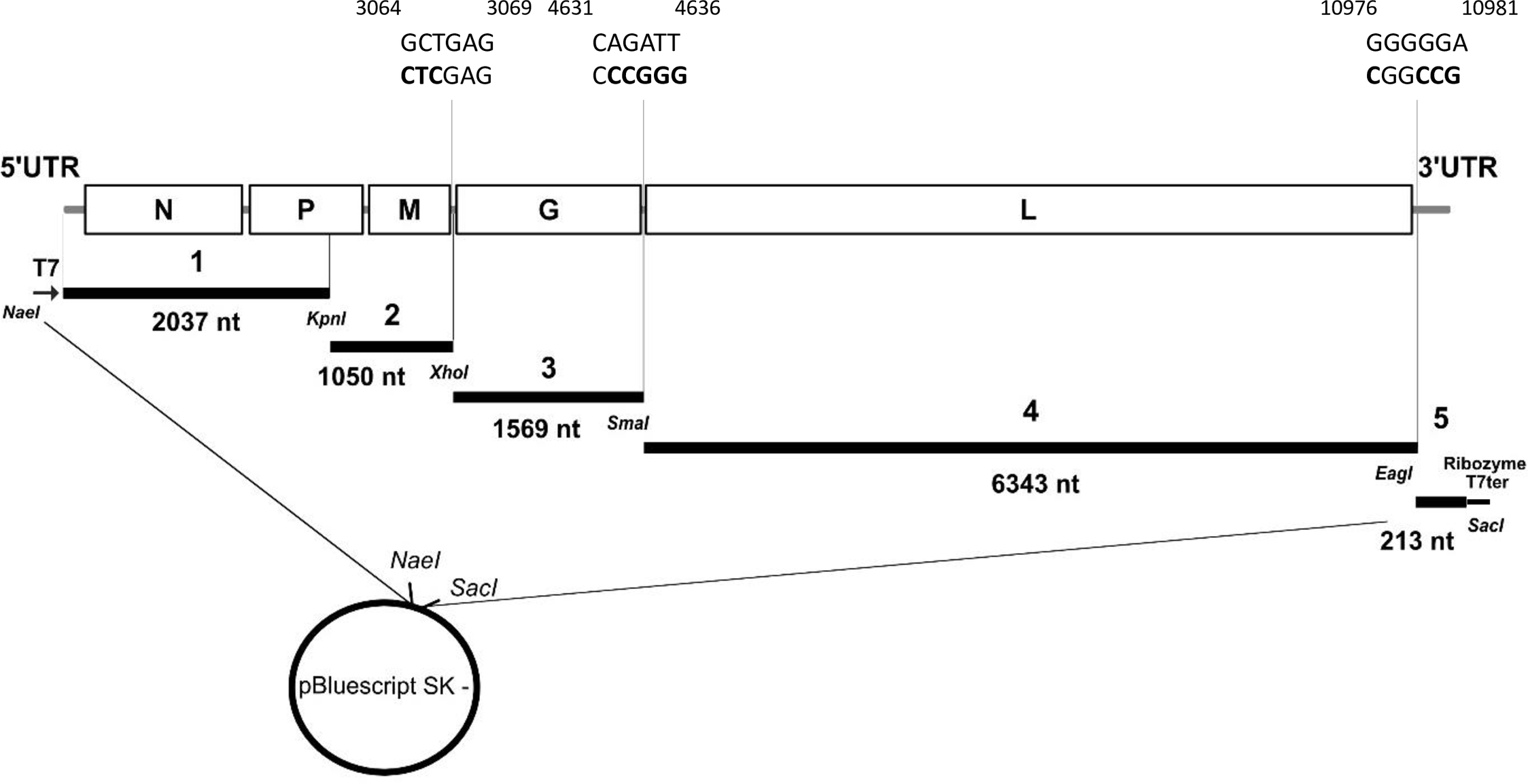

Supplement: S1 Fig — The details of the design of infectious SVCV cDNA are described in Materials and Methods. Shown are XhoI and SmaI sites that were introduced into the M-G, G-L intergenic regions, respectively and EagI site that was introduced in the trailer region: the sequence and nucleotide position of each site in the antigenome are indicated, with nucleotide substitutions made to create the sites in bold and the wild-type sequence shown above. (TIF) [file ppat.1012328.s001.tif]

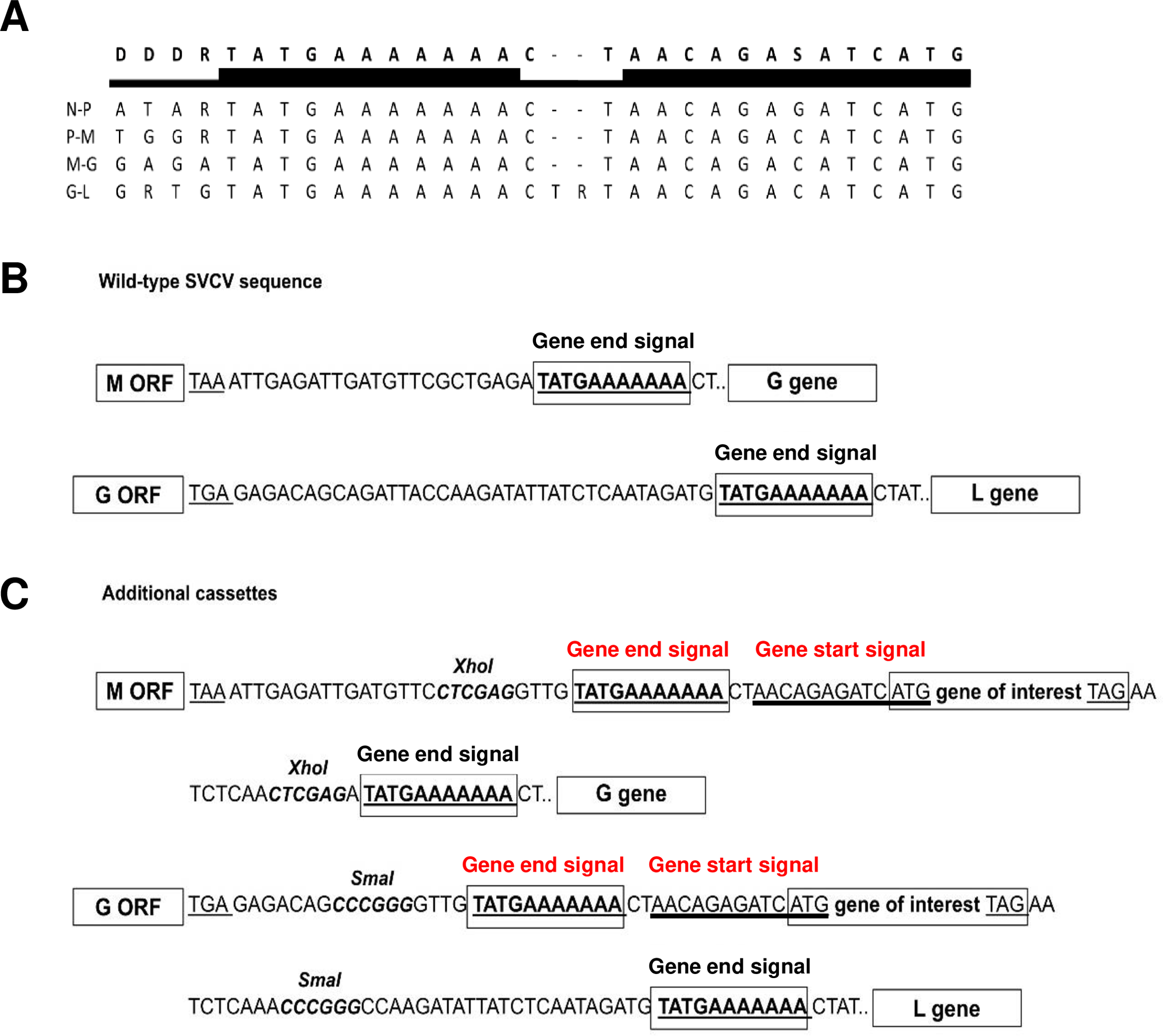

Supplement: S2 Fig — A. Alignment of SVCV intergenic regions. The complete intergenic regions between N-P, P-M, M-G and G-L genes of 16 SVCV strains were aligned to define the minimal consensus sequence DDDRTATGAAAAAAACTAACAGASATCATG (with D = G, A or T, R = G or A, and S = G or C). This untranslated region is composed of the transcriptional termination/polyadenylation gene end (GE) signal, TATGAAAAAAA, and the transcription initiation gene start (GS) signal, AACAGASATCATG (with S = G or C). B. Details of the intergenic regions between M-G and G-L genes in the wild-type SVCV sequence. Stop codons are underlined and gene end signals are boxed. C. Details of the insertion of an additional gene in the M-G and G-L intergenic regions with the creation of a XhoI and SmaI unique restriction sites, respectively. Additional GE and GS signals are indicated in red upstream of the additional gene (gene of interest). (TIF) [file ppat.1012328.s002.tif]

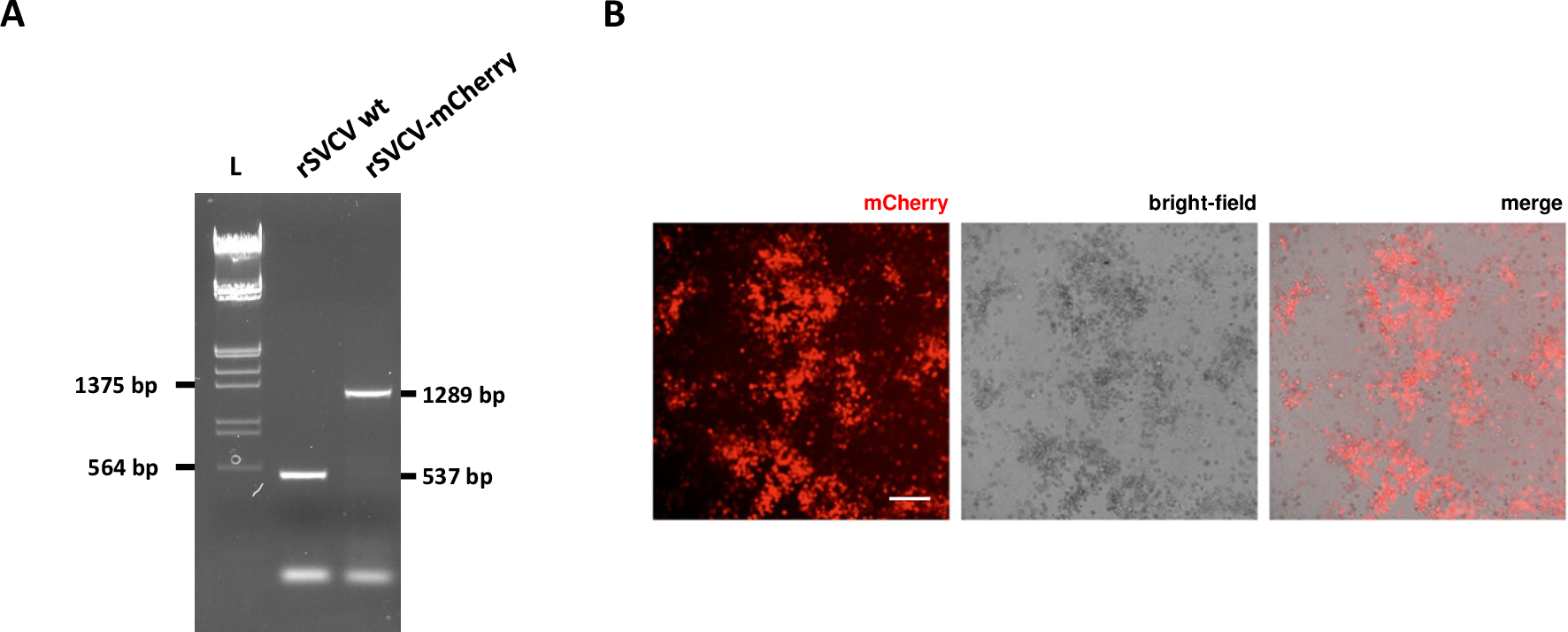

Supplement: S3 Fig — rSVCV-mCherry was passaged up to 10 times on EPC cells. At the tenth passage, viral RNA was extracted from infected cell supernatants to confirm the presence of the mCherry expression cassette and its sequence by Sanger sequencing (A). RT-PCR products amplified with specific primers (5_SvcvCasMG; CATCAACATGGATACAACGGGATGG and 3_SvcvCasMG; CATTCAGCTGCATGGCAGATCCATC) from wild-type rSVCV (rSVCV wt) and rSVCV-mCherry (Passage 10) were analyzed on a 1% agarose gel. The sizes of the bands of the ladder (L) and the specific PCR fragments are indicated on the left and on the right, respectively. rSVCV-mCherry positive EPC cells at passage 10 (B). The cells were incubated at 25°C for 32 hours. Live cell monolayers were then visualized with a UV-light microscope. Scale bars, 100 μm. (TIF) [file ppat.1012328.s003.tif]

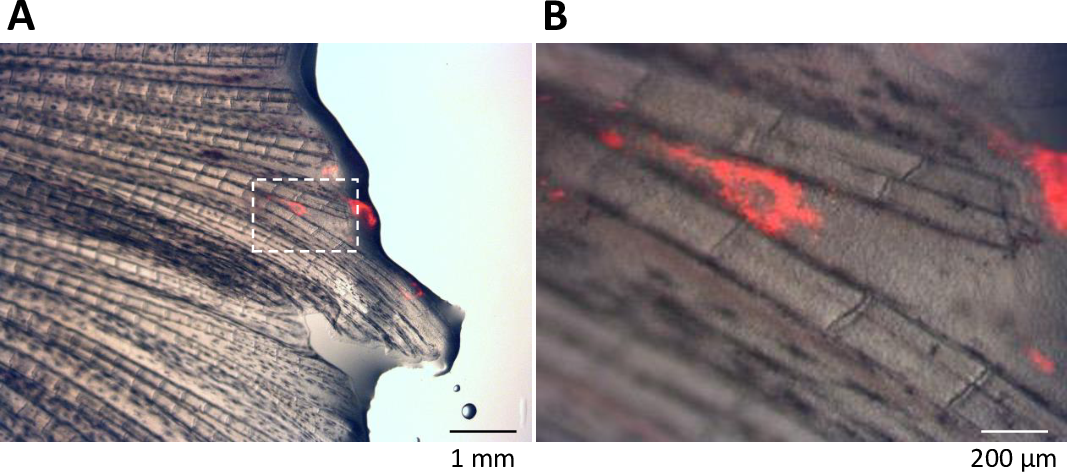

Supplement: S4 Fig — A. Visualization of rSVCV-mCherry at day 3 post infection by stereomicroscopy in the caudal fin of bath-infected carp. Infected foci (red) are located at the margin of the caudal fin and between bony rays (interray). Scale bar: 1 mm. B. Magnified view of inset depicted in A showing infection of interray. Scale bar: 200 μm. (TIF) [file ppat.1012328.s004.tif]

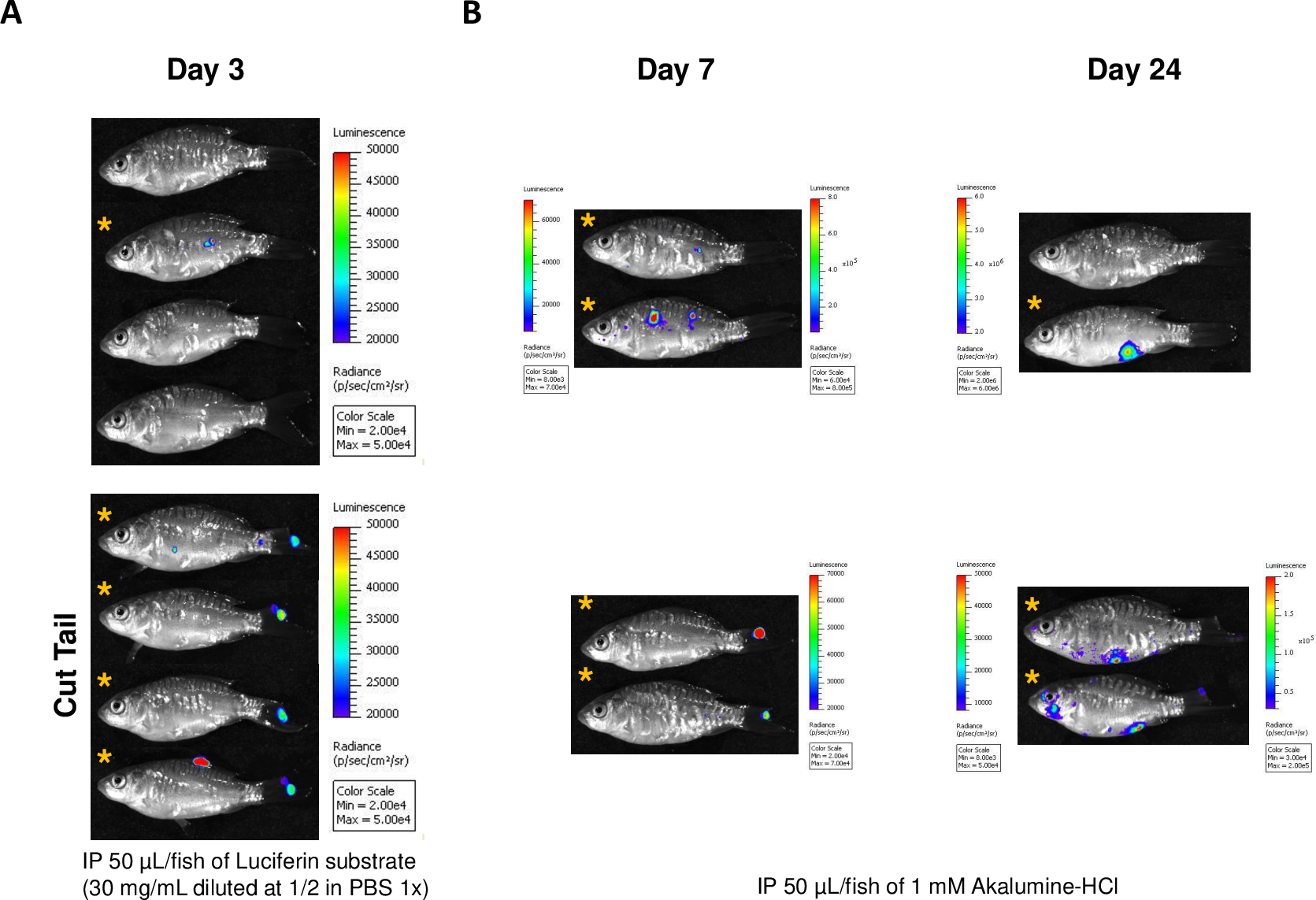

Supplement: S5 Fig — A. Carp (mean weight, 1.91 g) were divided into two groups with one group that had their caudal fins cut (cut tail) prior to infection by immersion with rSVCV-ffLUC G/L. At 3 days post infection, 4 fish in each group were randomly harvested, anesthetized, and IP injected with 50 μL of luciferin (30 mg/mL) before imaging using an IVIS Spectrum BL imaging system. Stars indicate carp with detectable bioluminescent foci. B. Carp (mean weight, 1.91 g) were divided into two groups as described above prior to infection by immersion with rSVCV-akaLUC G/L. At 7- and 24- days post infection, 2 fish in each group were randomly harvested, anesthetized, and IP injected with 50 μL of Akalumine-HCl substrate (1 mM) before imaging. Stars indicate carp with detectable bioluminescent foci. (TIF) [file ppat.1012328.s005.tif]

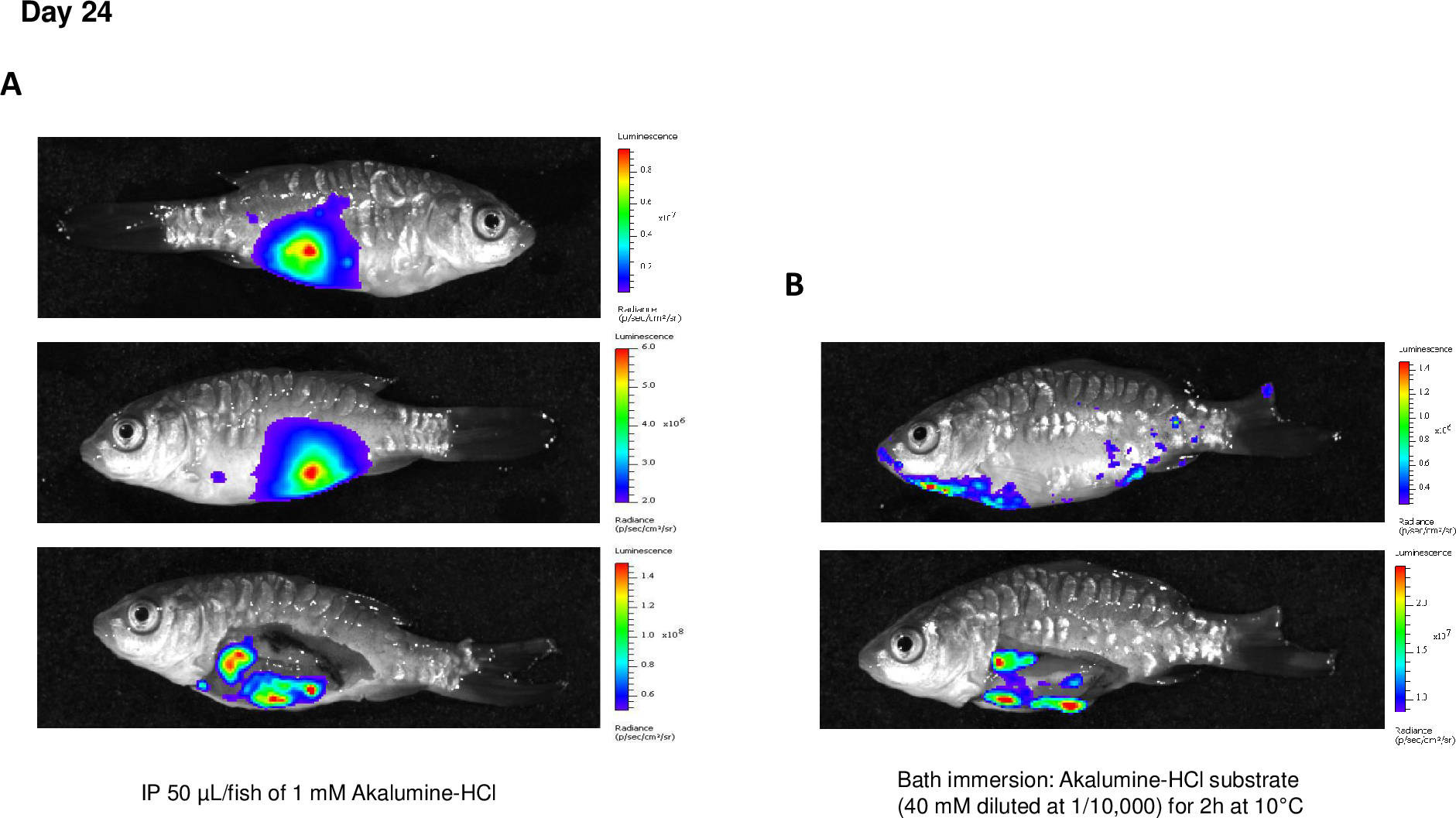

Supplement: S6 Fig — A. Delivery of Akalumine-HCl substrate by IP injection (50 μL/fish of 1 mM Akalumine-HCl). The same fish before (both sides) and after dissection is shown in panel A. B. In order to compare both bioluminescence profiles, the picture of the fish shown in Fig 4 and corresponding to the delivery of Akalumine-HCl substrate by bath immersion for 2 h at 10°C (40 mM diluted at 1/10,000 in water) was included in this supplementary figure. The same fish before and after dissection is shown in panel B. (TIF) [file ppat.1012328.s006.tif]

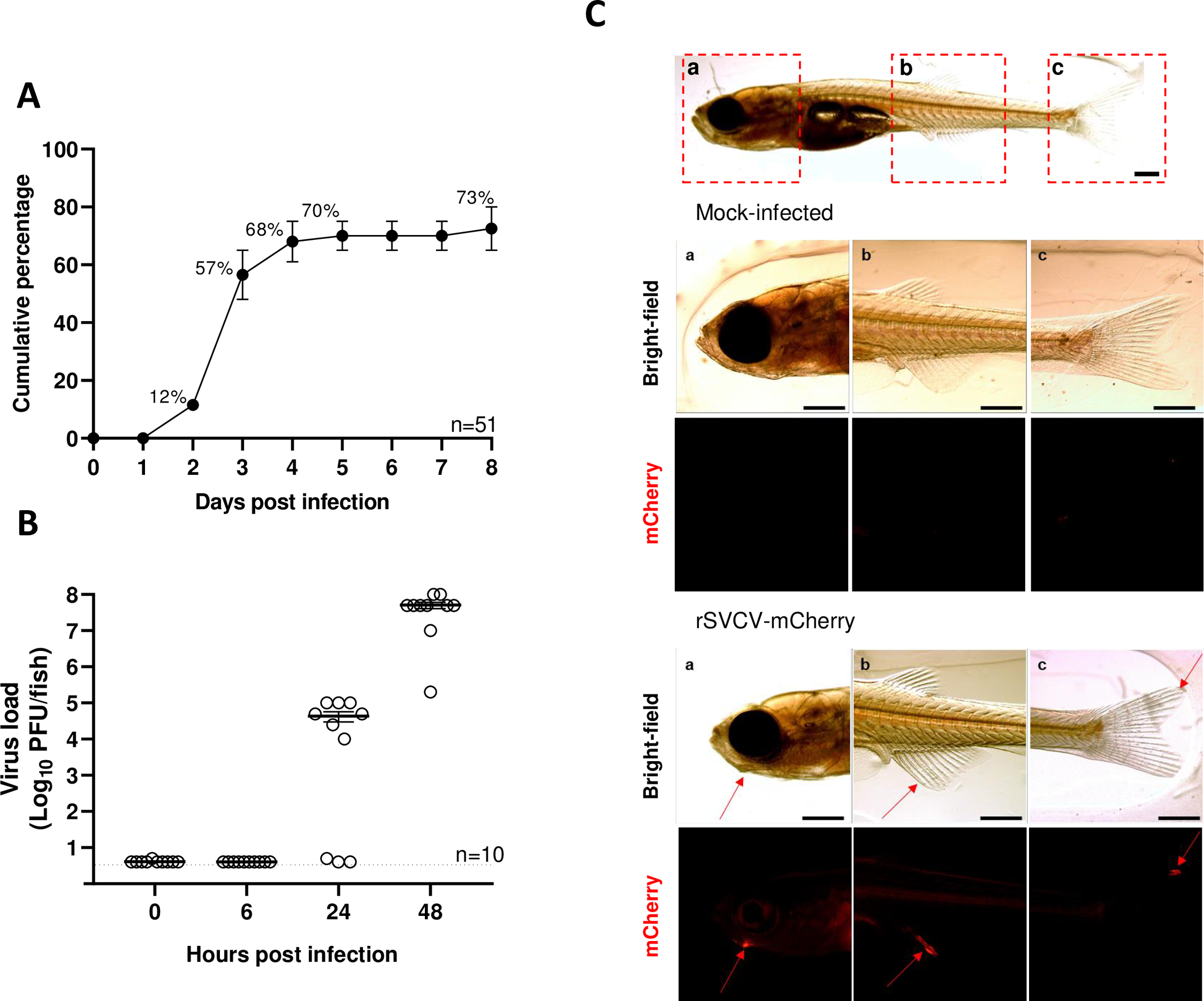

Supplement: S7 Fig — A. Nacre zebrafish juveniles (n = 51) at 25 dpf were infected by bath immersion with rSVCV-mCherry (1 × 10^7^ PFU) and incubated at 24°C. Mortality was recorded daily and is presented as the mean of the cumulative percent of dead larvae recorded in two independent experiments. No mortalities were recorded in the mock-infected group (n = 25). B. Virus load in individual juveniles. At different times post infection, juveniles (n = 10 in 2 independent infections) were randomly harvested and virus load was determined by plaque titration in EPC cells. Means are shown together with standard errors. C. Detail of mCherry fluorescent foci at 24 hpi in different infected juveniles. Pictures are presented for mock infected (top panel) and SVCV infected fish (bottom panel) in the following order: brightfield and red fluorescence. Red arrows indicate the areas of infection. Scale bars: 500 μm. (TIF) [file ppat.1012328.s007.tif]

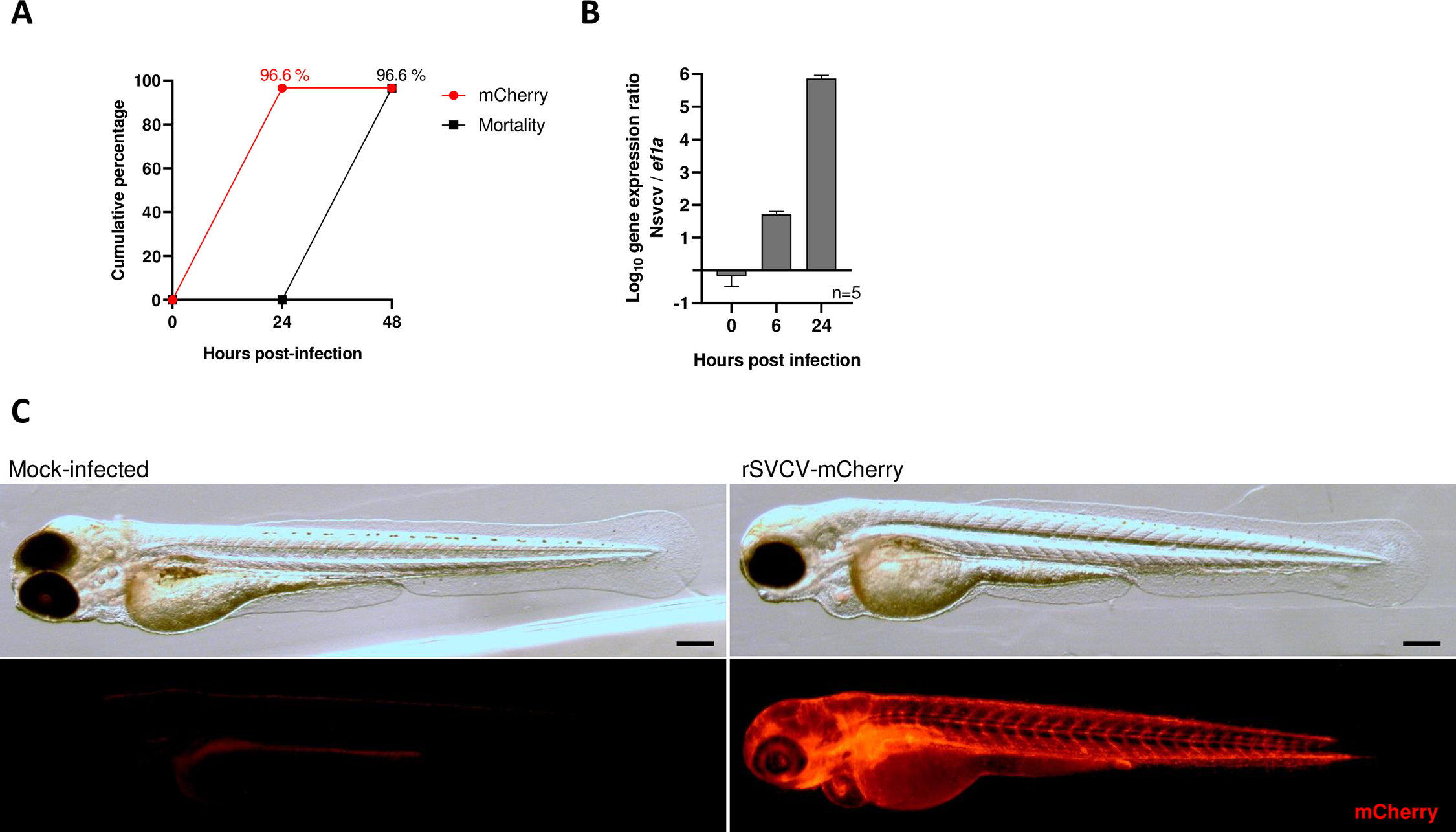

Supplement: S8 Fig — A. Mortality is presented as the mean of cumulative percent of dead larvae recorded as well as the percent of mCherry positive larvae. No mortality was recorded in the mock-infected group. B. rSVCV-mCherry replication in zebrafish larvae microinjected in the duct of Cuvier. 5 groups of 5 larvae were randomly harvested at different times after infection (0, 6, and 24 hpi) and gene expression was analyzed by RT-qPCR. Virus loads are expressed as ratio of mRNA copy of SVCV nucleoprotein to ef1a housekeeping gene. Means are shown together with standard errors. C. Examples of zebrafish larvae microinjected in the duct of Cuvier at 24 hpi. Micrographs of mock- and rSVCV-mCherry infected larvae are presented in brightfield and red fluorescence. Scale bars: 200 μm. (TIF) [file ppat.1012328.s008.tif]

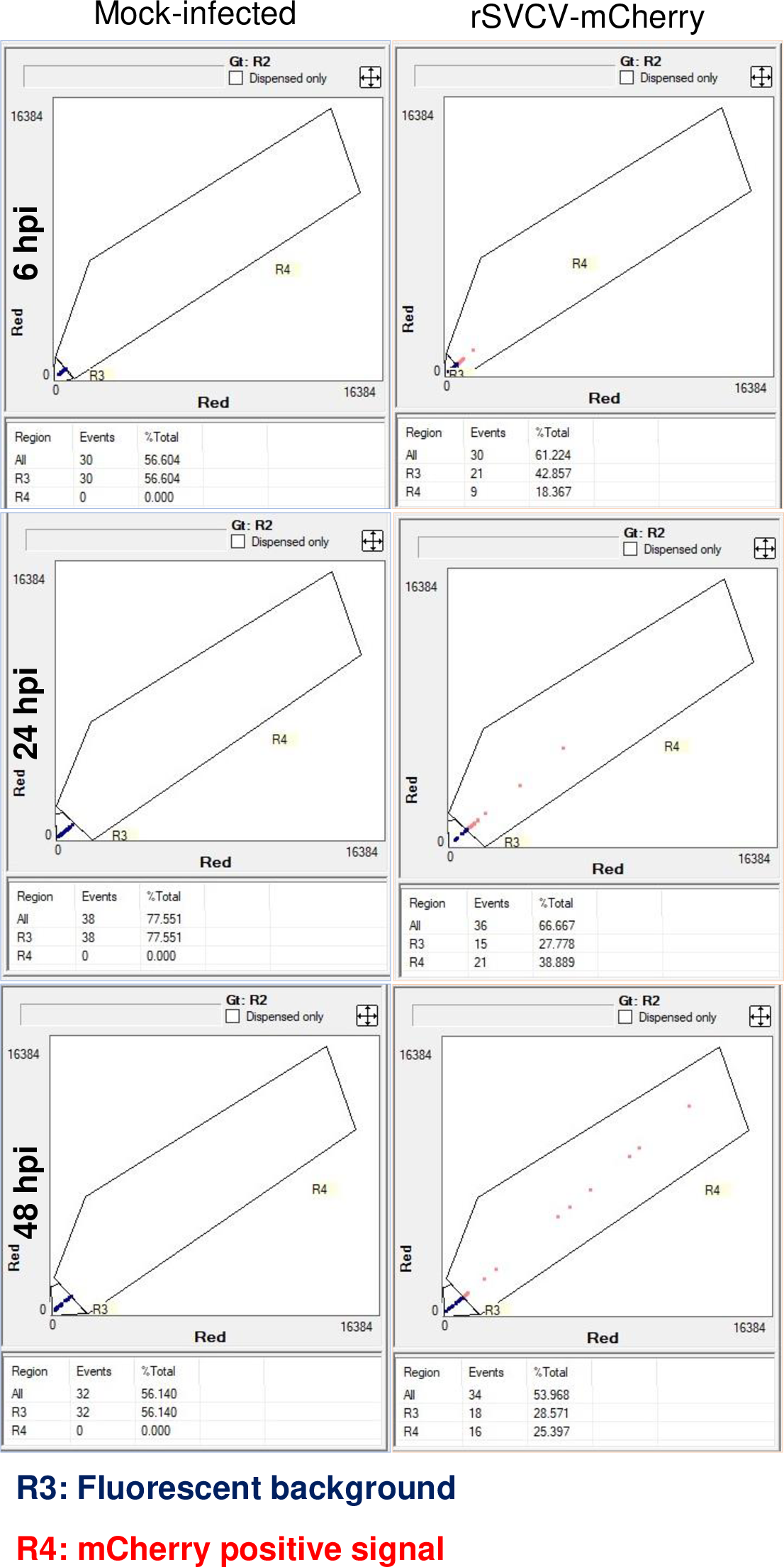

Supplement: S9 Fig — Dot plots of the red fluorescence signal of mock- and rSVCV-mCherry-infected larvae recorded with the COPAS system at different times post infection (6, 24, and 48 hpi). The R3 region was drawn around the mock-infected larvae to define the background signal for mCherry (autofluorescence) and the R4 region was delineated in rSVCV-mCherry infected larvae to determine the positive signal for mCherry. (TIF) [file ppat.1012328.s009.tif]

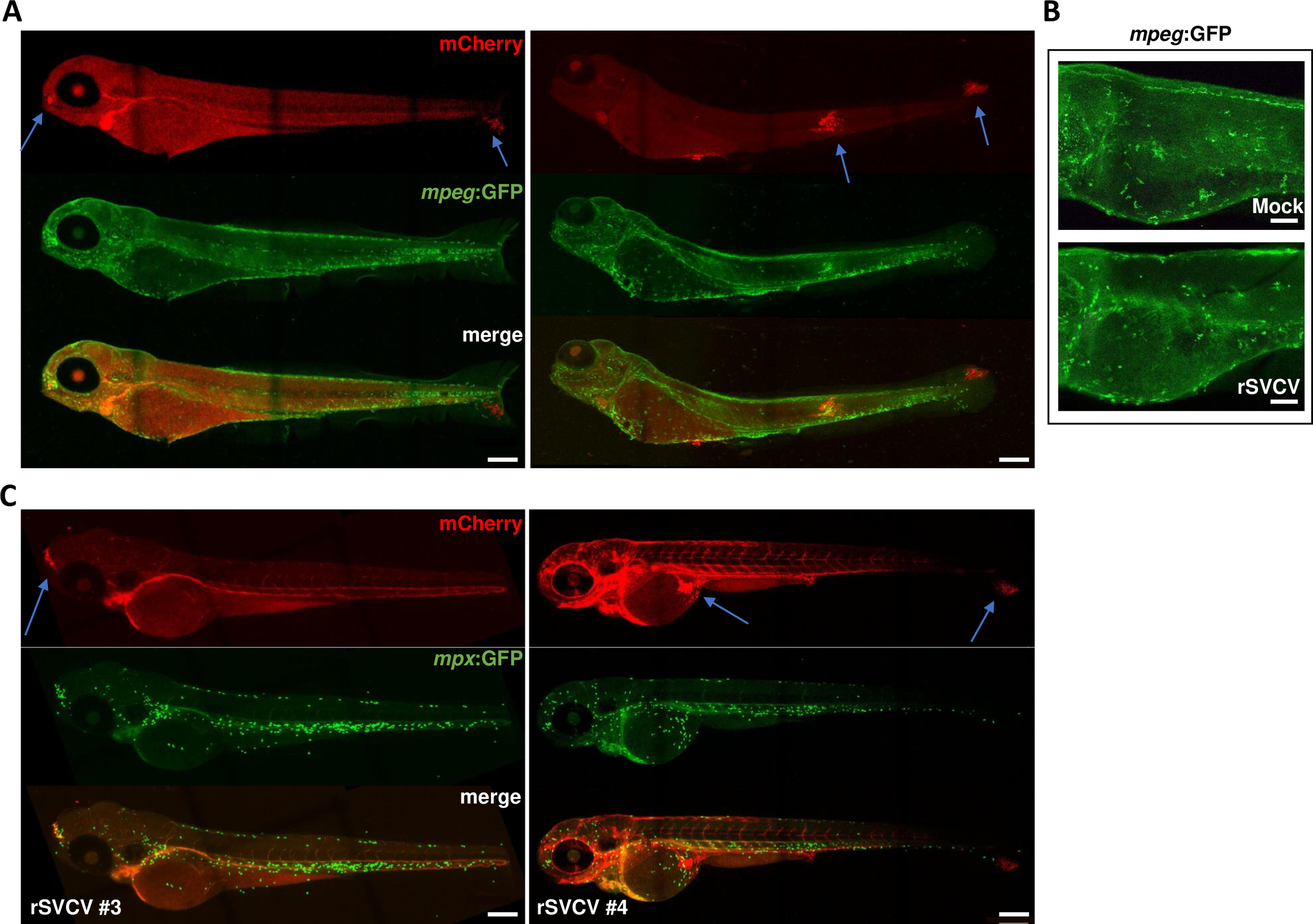

Supplement: S10 Fig — A. Visualization of macrophages (green) at 24 hpi by confocal microscopy in the Tg(mpeg1:eGFP)gl22 transgenic line infected with rSVCV-mCherry (red). Blue arrows show sites of viral replication. Scale bars: 200 μm. B. Area of the trunk of Tg(mpeg1:eGFP)gl22 transgenic larvae in which mock-infected larvae macrophages show a typical ameboid shape and rSVCV-infected larvae macrophages have rounded shape even in an area located far from the virus replication sites. Scale bars: 100 μm. C. Visualization of neutrophils (green) at 24 hpi by confocal microscopy in the Tg(mpx:GFP)i114 transgenic line infected with rSVCV-mCherry (red). Blue arrows show sites of viral replication. Scale bars: 200 μm. (TIF) [file ppat.1012328.s010.tif]

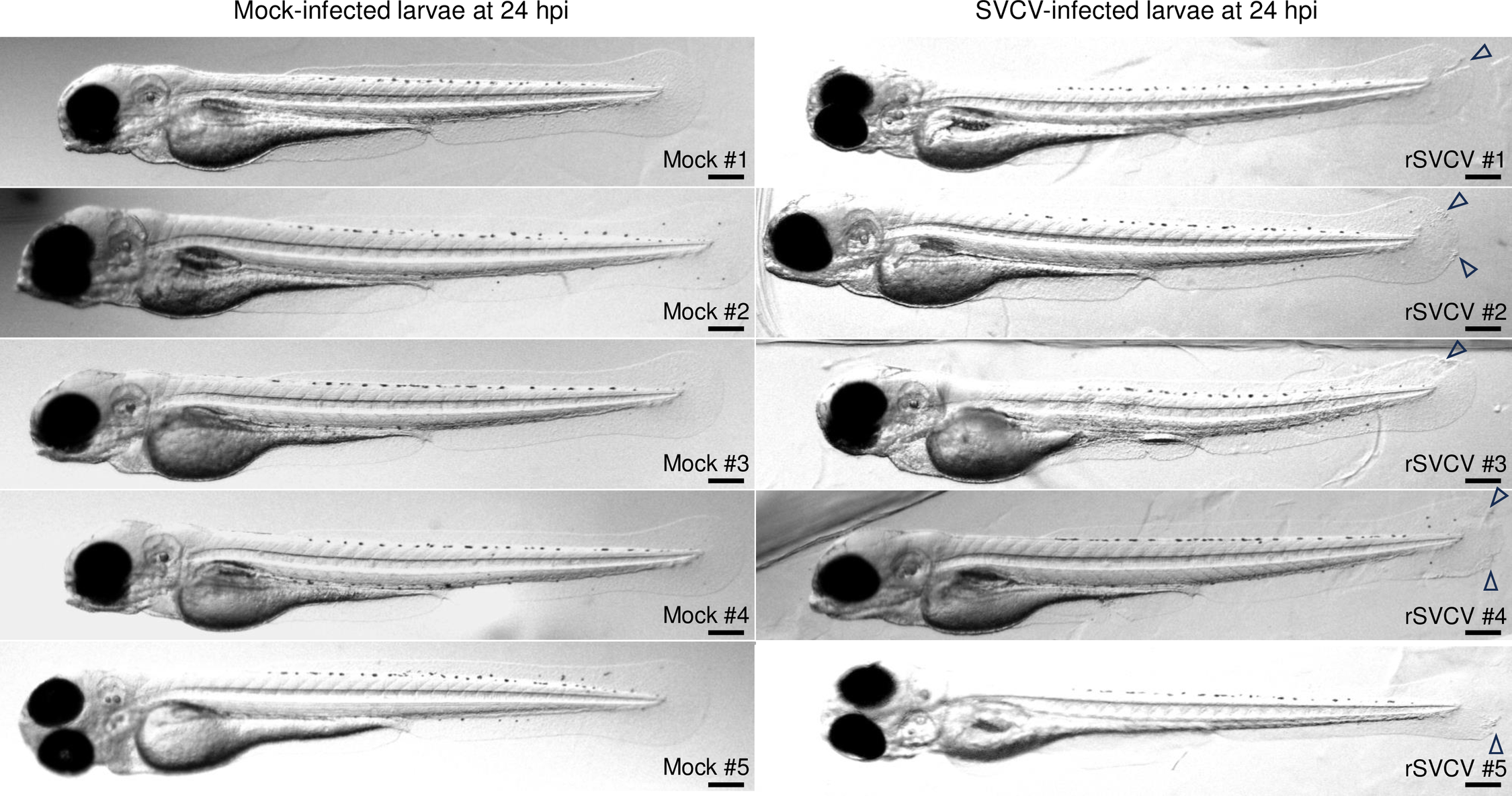

Supplement: S11 Fig — Micrographs of mock- and rSVCV-infected larvae at 24 hpi taken with a stereomicroscope. Micrographs in brightfield with modified contrast to perceive the fin fold. Head arrows point to small wounds in the SVCV-infected larvae that were not observed in mock-infected larvae. Scale bars: 200 μm. (TIF) [file ppat.1012328.s011.tif]

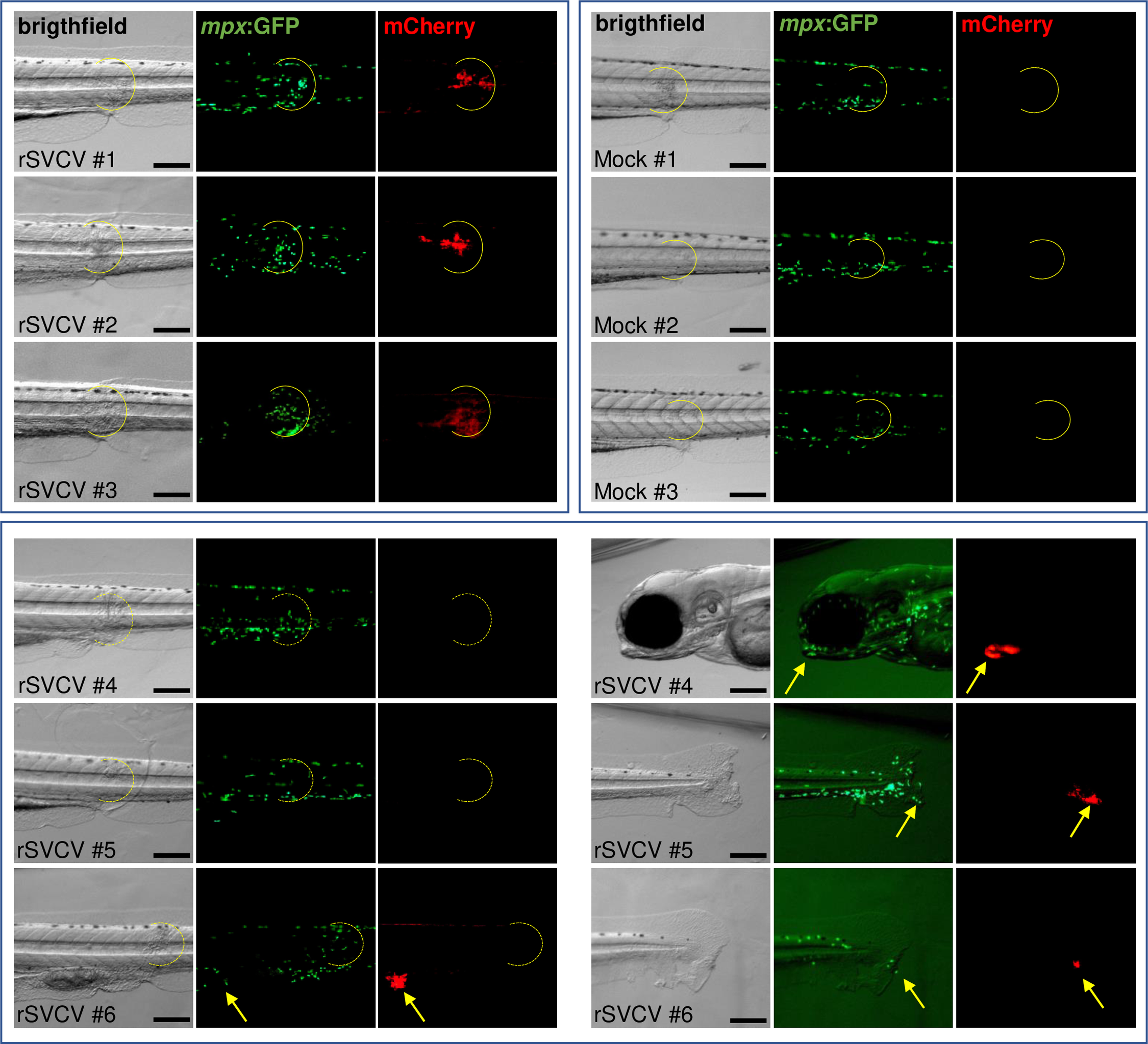

Supplement: S12 Fig — Brightfield and fluorescence microscopy images (mpx:GFP, neutrophils in green; mCherry, rSVCV in red) of SVCV- and mock-infected Tg(mpx:GFP)i114 larvae at 26 hpPA and 24 hpi. The photoablation area is marked with a yellow dotted crescent, and infected sites far from this area are pointed with yellow arrows. For rSVCV-infected fish #4–6, other primary sites of infection are shown. Scale bars: 200 μm. (TIF) [file ppat.1012328.s012.tif]
