# Supplementary material for: In vivo multiscale analyses of spring viremia of carp virus (SVCV) infection: From model organism to target species
Source: PLoS Pathog. 2024 Aug 5;20(8):e1012328. doi: 10.1371/journal.ppat.1012328 (PMC11326706; doi:10.1371/journal.ppat.1012328)
Supplement: Attachment — Submitted filename: Responses to reviewers.docx [file ppat.1012328.s021.docx]

The design and development of recombinant Sprivivirus cyprinus/SVCV strains and the tracking of the bioluminescent virus in infection models (both zebrafish and natural host) in vivo leads to key biological observations, notably about the entry pathway of the virus. The model appears elegant as it provides a unique opportunity to visualize in real time the virus in infected cells and to analyze the subsequent immune response.

Nonetheless, authors should address the main points raised by reviewer 1, namely about the genetic stability of the recombinant strains that are produced and about the absence of virus detection in some tissues. Some additional controls are mandatory to answer these points. Answers to the minor points addressed by the two reviewers should also be provided.

Dear Editors,

We would like to thank you for your positive remarks on the manuscript. In the current letter, we have considered and responded to every specific point addressed by the reviewers. We have modified the manuscript accordingly as described in detail in the responses to reviewers and highlighted in the revised manuscript. The additional experiments and the new supplemental figures/video helped to clarify the results on the main points and to improve the quality of our manuscript which we hope is now suitable for publication in Plos Pathogens.

Reviewer's Responses to Questions

**Part I – Summary**

Reviewer #1: In this manuscript Souto et al reported the first reverse genetics system for Sprivivirus cyprinus (previously called Spring viremia of carp virus (SVCV)), the use of this system for production of recombinant viruses expressing reporter genes, and the characterization of the infection by some of the recombinants using two in vivo infection models (common carp and zebrafish). This study led to key biological observations among which the most interesting is probably the identification of the skin epidermis as the major portal of entry of SVCV into its host. This study provides one more evidence that the skin epidermis of teleost fishes acts as a major portal of entry of viruses belonging to distant phylogenetic classes. This study represents a significant amount of data that will interest the scientific community working on fish infectious diseases and using zebrafish as a laboratory model. Few key experiments must be performed to support the conclusions of this MS. Other minor aspects of the MS should be addressed to further increase its quality.

Reviewer #2: Spring viremia of carp virus (SVCV) is an important fish virus that is a threat to aquaculture and ecosystems. The authors describe their work in developing recombinant SVCV strains that express fluorescent and luminescent proteins that enable tracking of viral infections. With these rSVCV strains, the authors demonstrate the arc of SVCV infection using immersion models in carp and immersion and intravenous injection models in zebrafish. The authors replicate aspects of natural infection, and thus the infection models they developed have great potential in understanding the course of SVCV infection and the immune response fish species mount to these infections. In addition, these infection models may help develop therapeutic strategies that may confine and/or eliminate SVCV infections. The work described in this manuscript is significant and has the potential to move the field forward. Overall, the general execution and scholarship is good, save for a few concerns about how the data are presented and the controls used in some experiments.

We would like to thank the reviewers for their positive and pertinent remarks on our manuscript. Our responses to each specific question are presented below.

**Part II – Major Issues: Key Experiments Required for Acceptance**

Reviewer #1: 1/ A key point in this study that needs to be addressed is the genetic stability of the recombinant produced when passed in cell culture or in vivo. Indeed, the present study demonstrated that insertion of reporter sequence into the genome of the parental strain led to a reduction of viral fitness (Figure 3). Consequently, mutations leading to the loss of the reporter sequence would inevitably lead to the selection of such mutants in the population of virions. Of course, such mutants would be “invisible” as they do not express the reporter gene but they could affect the biological observations through “quasi-species” phenomena. It is also possible that transcomplementing mutations could be selected by passage in vitro and/or in vivo. The kinetics of mortality reported in Figure 3 for the recombinants supported these hypotheses. Of note, while the virulence of the rSVCVffluc recombinants does not seem to be affected by the insertions based on final mortality rates, it is astonishing that the appearance of the first dead fish occurred after 3 to 4 times longer incubation period compared to the parental control. This result could be explained by progressive selection of mutants according to time.

To address these hypotheses, the present study must address genomic (sequence) and phenotypic (reporter expression, viral replication in cell culture (growth, plaque size)) stability throughout passages both in cell culture and in vivo (fish infection).

We thank the reviewer for this important point on the genetic stability of the recombinant SVCV viruses.

**Mutations leading to the loss of the reporter sequence:**

With the exception of a single report on respiratory syncytial virus (Spann KM, et al. JVI 2003 DOI: [10.1128/jvi.77.20.11201-11211.2003](https://doi.org/10.1128/jvi.77.20.11201-11211.2003)), genetic recombination in the order *Mononegavirales* has not been reported, thus appearing to be a very rare event. We agree that single mutations in the reporter gene could render the virus “invisible”. However, for detection of the primary replication site, we focused on early infection events with a minimal risk of loss of reporter gene expression. Moreover, at later time point of infection (24 days post infection) Figure 4D shows that the reporter gene is still highly expressed and strong bioluminescent signals are detected in previously described target organs from infected fish. Another evidence comes from re-isolation of rSVCV-mCherry from infected zebrafish (larvae in Fig. 5C and adults Suppl. Fig. 3B) that was still expressing the mCherry reporter in the viral titration assays. Finally, we further addressed the question of the reviewer by performing new experiments to evaluate the genetic stability of the rSVCV-mCherry *in vitro.* After performing 10 iterative passages in cell culture, the reporter gene is still present and contains no mutations and the expression of the mCherry remained intact, reinforcing our data to demonstrate the stability of such a traceable virus (Suppl. Figure 3).

**Invisible viruses could affect the biological observations**: In zebrafish, biological observations are based on *in vivo* observations of mCherry signals. The increase of mCherry signal intensity over time post infection was demonstrated by fluorescence imaging (Figure 5A, 6B), virus titration (Figure 5C, 8B); rtqprc (Figure 5D), and flow cytometry (Figure 8A). Importantly, we showed that the increase in mCherry signal intensity correlates with the increase in infectious titer over time (Figure 8C, r^2^=0.9262), in favor of a limited effect of invisible viruses on biological observations. In addition, the presence of invisible (mCherry negative) viruses was assessed by *in toto* immunochemistry and tissue clearing of infected larvae performed with anti-N antibodies (Figure 5B). Confocal images show that the expression of mCherry fluorescent protein was correlated with the expression of the viral nucleoprotein (Nsvcv) in co-immunolabelled larvae supporting a limited proportion of invisible viruses (Figure 5B).

2/ Another key point to keep in mind when using bioluminescent or fluorescent reporters is that “what you detect is real but it is possible that the presence of the virus is undetectable in some tissue for different reasons (access of substrate, expression of the reporter, access of excitation light in case of fluorescence …)”. When using bioluminescent reporters, two obvious points must be addressed. Does the substrate reach all tissues? Is there any structure preventing the light emitted by the tissues to reach the detector? In relation to these two questions, the present study will benefit from data resulting from injections of the luciferase substrates (both) and the use of the recombinants expressing the two different luciferases used. Also, ex vivo bioluminescent analyses directly exposing organs to the detector are mandatory before concluding that they are negative for viral replication. This comment is particularly important for gills.

In the present work, we described a technological development based on the ffLUC-derived luciferase AkaLUC, a novel luciferase reporter with more than 100-fold higher sensitivity for *in vivo* tracking of infected cells as compared to the standard ffLUC [22] combined to a highly deliverable luciferin analog [23] amenable to detect infectious foci of rSVCV-Luc by immersion of the fish in the substate instead of injection.

**Does the substrate reach all tissues?** To support the results presented in Figure 4 and answer the reviewer’s concerns, we added a new supplementary Figure X2A established from data not shown in the previous version comparing the delivery route of the substrate (bath versus injection) for standard ffLUC and AkaLUC reporter viruses.

First, we compared bioluminescence profiles obtained after infection with ffLUC and akaLUC virus encoded luciferase reporters. Fish display similar profiles of bioluminescence emission at day 3 post infection when infected with rSVCV-ffLUC, IP-injected with luciferin compared to that infected with rSVCV-akaLUC, bathed in Akalumine-HCl substrate (Supplementary Figure 4A versus Figure 4).

Supplementary Figure 4B shows that carp infected with rSVCV-akaLUC but IP-injected with Akalumine-HCl substrate have identical bioluminescence profile to infected ones bathed in Akalumine-HCl substrate at day 7 and 24 post infection (Figure 4). Examination of bioluminescent signals in internal tissues, exposed upon dissection revealed that the Luc expression profiles were equivalently detected at day 24 post infection irrespectively of the route of luciferase substrate administration, indicating proper overall distribution of the substrate for both routes of delivery (Supplementary Figure 5).

Thus, Figure 4 and supplementary Figures 4-5 demonstrate that the observed viral tropism of rSVCV in infected carp is not dependent of the LUC reporter or the administration route of the substrate. The text has been edited to present the comparative analyses and the conclusion.

**Is there any structure preventing the light emitted by the tissues to reach the detector?**

We agree with the reviewer that some structures can prevent the light emitted by the tissues to reach the detector. To overcome this limitation, we used Akaluciferase and the luciferin analogue AkaLumine-HCl to develop a more sensitive method for detecting *in vivo* virus infectious foci in fish species.

While Akaluciferase has a higher signal strength than standard ffLUC, Akalumine-HCL has been screened over water-soluble derivatives of AkaLumine and selected based on its light emission ranges in the NIR wavelength ranges (*λ*_max_=677 nm) beyond the visible light spectrum, in which haemoglobin (*λ*=∼415–577 nm) and melanin (*λ*<600 nm) dominate absorption. This allows the optimization of acquisition parameters to increase bioluminescence production and detection sensitivity from deep-tissue targets at very low concentrations.

This powerful Aka-bioluminescence imaging was described as extremely sensitive and enabled the visualization of a single bioluminescent cell present in deep tissues of mice and monkeys [22]. Measurement of bioluminescence signals from internal tissues, potentially undetected by the detector, was also carried out after dissection in order to assess the overall signals emitted by the infected cells in the fish body (Supplementary Figure 5).

When using this extremely sensitive approach, no bioluminescent foci could be detected in the gills of any of the fish analyzed as part of our study. This phenotype (absence of primary replication foci in the gills) was confirmed in waterborne zebrafish model of SVCV infection at larvae and adult stages.

3/ Concerning the demonstration that the skin acts as the major portal of entry of the virus into its host, it will be very interesting to demonstrate at the subcellular level that epidermal cells are indeed subject to viral infection. A former study on viral entry into fish adopted this approach by electronic microscopy taking profit of the bioluminescent signal to target sample collection. Similarly, observation in zebrafish should be further supported by data demonstrating at the histological level the infection of the epidermis.

Based on the observations that the skin and fins act as the major portal of entry in carp infected with rSVCV-akaLUC (Figure 4), we further investigated the infection of epidermal cells at the subcellular level. Fluorescence imaging of carp tail fins in live and fixed tissues (immunohistochemistry and tissue clearing) were carried out from fish infected by immersion in rSVCV-mCherry (new supplementary Figure 4 + supplemental video 1).

These data were confirmed by evidence obtained from zebrafish experiments conducted on *Tg(il1β:GFP-F)* transgenic larvae expressing GFP fluorescence in keratinocytes (easily recognized by their polygonal shape) at the tip of the caudal fin, in the retina, and neuromasts in naïve fish and in IL1b producing cells upon stimulation [26]. Indeed, confocal 3D imaging presented in supplementary Video 2 showed the presence of mCherry and GFP double positive keratinocytes.

Reviewer #2: There are a few figures that lack control data, including figure 5 and supplementary figure 3. It seems that it would be appropriate to show negative control data within these figures. While figure 2 shows that mock infection does not cause any mortality, mock infection data should be shown in figure 5 and supplementary figure 3.

We thank the reviewer for this comment. The control data have been included in Figure 5 and supplementary Figure 3 and the figure legends have been edited accordingly.

**Part III – Minor Issues: Editorial and Data Presentation Modifications**

Reviewer #1: 1/ Spring viremia of car virus (SVCV) is the common name used to describe the virus subject of the present study. However, this name has been changed by the ICTV in 2015 to Sprivivirus cyprinus (<https://ictv.global/taxonomy/taxondetails?taxnode_id=202201765&taxon_name=Sprivivirus%20cyprinus>). It seems important to his reviewer to mention or even to use the official name of this virus in this MS. To provide the best visibility to this MS, it is suggested to mention both the former and current virus nomenclature in the MS but also in related links.

Done. “*Sprivivirus cyprinus*, commonly known as spring viremia of carp virus (SVCV), belongs to the *Sprivivirus* genus in the *Rhabdoviridae* family.”

2/ Throughout the MS, there are repetitions that should be deleted. This is particularly the case of the discussion which repeats sections of the results.

The discussion section has been reduced to avoid repetitions of the results.

3/ This MS will benefit from a systematic presentation of all the recombinant (structure and data). See for example figure 2A which presents only three of the recombinants produced.

All five genome constructs have been added in fig. 2A. The legend of this figure has been modified accordingly.

4/ At the beginning of the results (Figure 1), the authors justify sequencing and phylogenetic analyses as the basis for the rational selection of the Fijan strain as the parental strain for construction of the recombinants (see line 139-142). “To select a representative genome sequence for establishing a robust reverse genetics system …”. Surprisingly, this strain appears distant from most strains which share a monophyletic origin. Consequently, this section sounds paradoxical to the reader. It must be modified.

To address this issue, we have separated the rationale behind the choice of using the prototype Fijan strain for reverse genetics from the phylogenetic/sequence analyses. We have added an explanation for the apparent phylogenetic distance between Fijan and the other more recently sampled SVCV genomic sequences. The corresponding paragraph has been modified accordingly. We hope that in its revised form the paragraph avoids any confusion and appears clearer to the reader.

5/ Line 141,171, the authors seem to quote inconsistent numbers of genome sequences, 16 versus 14. Please double check and correct if necessary. In case the numbers are correct, please provide a short explanation in the MS.

You are right both analyses were performed with the 16 SVCV genome sequences.

6/ Figure 7B. It will be interesting to present the data using 3D graphics so that correlations between expression could be established (e.g. are the fish positive at the head systematically positive for the tail …).

We have taken into consideration this point by replotting the data of Figure 7B using a 3D graphics representation (see figure provided below). However, because such representation does not allow to include error bars and makes it difficult to place the larvae counts for each condition, we decided to keep the original 2D representation as it allows to better display the full scope of data analyzed for this experiment.

Fig. 7B using 3D graphics representation.

7/ Figure 9. Is it possible to provide the data for fish inoculated by IV and analysed at 48hpi?

At 48 hpi all the larvae are dead (see Suppl. fig. 4).

Reviewer #2: Overall, the manuscript is written well, but there are several issues with grammar and syntax, including punctuation, missing words, and word choice. This reviewer respectfully asks the authors to better edit their manuscript. Perhaps they could consider working with a scientific copy editor.

The manuscript was edited.

The introduction is quite expansive and could be made more concise.

PLOS Pathogens guidelines indicate that Length manuscripts can be any length. There are no restrictions on word count, number of figures, or amount of supporting information.

Additional comments follow:

Line 35: missing word in the sentence?

“us” was added

Line 57: missing word in the sentence?

“ the viral spreading and **the** target organs…”

It would be interesting to see the three nucleotide mutations annotated in figure 1B.

The three nucleotide substitutions were mapped on Figure 1B and details on nucleotide substitutions to create the additional enzyme restriction sites were added in supplementary Figure 1. The legend of these two figures has been modified accordingly.

It would be interesting to see the sequence alignment of the 16 SVCV strains with features annotated, perhaps included in the supplementary information.

Panel A in supplementary Figure 2 summarizes the variability of the complete intergenic regions of the 16 SVCV genomic sequences. The legend of this figure has been modified to make this point clearer.

In Figure 2B, it would be helpful to see all five pSVCV constructs rather than just the three that were shown.

All five genome constructs have been added in Figure 2A. The legend of this figure has been modified accordingly.

Line 197-198: This sentence is confusing as to what was done. Can this be clarified? E.g., how long were they incubated after transfection?

As mentioned in the Materials and Methods section (line 718), the cells with the transfection mixture were incubated for 6 to 12 h at 37°C and the medium was then changed. The cells were incubated at 25°C for 7 additional days. The sentence has been modified.

Line 244: is it 2 out of 4 fish as 3 dpi, as it appears to be 3 out of 4 in the corresponding figure?

We agree 3 out of 4 fish were positive for SVCV infection but only 2 had SVCV replication foci at the tail cut site.

Line 244-245: “out to” could be replaced by “of.”

Done.

Line 258: “privileged” does not seem to be the correct word choice.

We have replaced it by “favored”.

Line 315: Please include references for this infection method.

The reference [26] has been included at the end of the sentence describing intravenous injection in the duct of Cuvier.

Line 349-350: Authors should consider applying the appropriate gene names for il1b and tnfa.

Done.

Lines 352-355 Was the expression of other zebrafish interferons considered? See Aggad et al or Varela et al.

Aggad *et al.* (10.4049/jimmunol.0901495) identified four IFNf genes, characterized in zebrafish model of SVCV infection (intravenous injection in the caudal vein described in Levraud *et al.*, 10.4049/jimmunol.178.7.4385). They showed that SVCV induced *IFN*f*1* and *IFN*f*4* in the embryo without modulation of *IFN*f*3* while *IFN*f*2* is only expressed in adult. In another study similarly based on intravenous injection (duct of Cuvier), Varela et al [26] showed that SVCV infection triggers *IFN*f*1* and *IFN*f*2* expression as well as *mxab* interferon stimulated gene.

In contrast, waterborne infection model of SVCV revealed inability of zebrafish larvae to mount an IFNf immune response despite the presence of a functional antiviral system (López-Muñoz et al., [10.1016/j.dci.2009.12.015](https://doi.org/10.1016/j.dci.2009.12.015)).

Hence, we examined the antiviral interferon signaling pathway: *IFN*f*1* expression, commonly described by Aggad and Varela and *isg15.*

Line 367: Mention transparency here. Other places, translucency is mentioned. Authors should pick one and be consistent.

We have replaced “translucency” by “transparency”.

Line 438: “impedes” may not be the best word choice.

We have replaced it by “interferes with”.

Suppl. Fig 6B: Is there a way to quantify the shift in macrophage morphology? It could be an even more powerful way of representing these data.

It would be possible to quantify the change in macrophage morphology in response to infection using imaging datasets, however this phenotype is not new and has already been described in Varela's study [26], which showed a change in the shape of macrophages from thin and elongated to rounded and lobulated morphology in SVCV infected fish.

Line 588-589: Authors show reduction of mpeg1 expression, not macrophage depletion directly. Do they have a direct method to test depletion (e.g., FACS)?

The reviewer is right and the text has been edited accordingly : As previously demonstrated [26], we showed that SVCV infection triggers **a reduction of the expression of macrophage marker** (Fig. 9E-F).

The y axis scaling for many of the panels in figure 9 is distorted. It appears that panels in figure 9A, 9C, and 9D can be rescaled for 20 to <5.

Graph axis have been scaled in order to allow easy comparison between both infection routes.

Line 542. Author should consider writing out full gene names for nfkb2 and relb rather than the shorthand they appear to use.

Done.

Line 599: Typo? “te”

Corrected.

Line 607: “impedes” may not be the best word choice.

We have replaced it by “interferes with”.

Line 614: Spelling of “correlated.”

Done.
